# Economic burden of ventilator associated pneumonia in a developing country

**DOI:** 10.1186/1753-6561-5-S6-P65

**Published:** 2011-06-29

**Authors:** E Alp, G Kalin, R Coskun, M Sungur, M Guven, M Doganay

**Affiliations:** 1 Infectious Diseases and Clinical Microbiology, Erciyes University, Kayseri, Turkey; 2 Erciyes University, Kayseri, Turkey

## Introduction / objectives

There is limited data about the economic burden of ventilator associated pneumonia (VAP) in developing countriesTo investigate incidence, risk factors, etiological agents, antimicrobial susceptibility rates and economic burden of VAP in a medical intensive care unit (MICU) of a developing country.

## Methods

All patients on mechanical ventilation were followed up during one year in MICU.

## Results

During the one-year period, 159 patients were followed up.Mean age was 61.82±16.81. VAP developed in 96 (60%) patients with 37.2/1000 ventilation days.The mean APCAHE II score was 24.32±5.94, and there was no difference between VAP and non-VAP patients.Median mechanical ventilation days for non-VAP patients were 3 days (1-15 days).Mortality rate for non-VAP patients was 88.5%, and the median time for death was 4th day of hospitalization.Median time for VAP development was 5.5 days (2-25 days).*Acinetobacter baumannii* (42%) and *Pseudomonas aeruginosa* (20%) were the most common pathogens.All microorganisms were multi-resistant.Imipenem resistance of *A.baumannii* and *P.aeruginosa* was 92% and 71%, respectively.The most significant risk factors for VAP were stay in hospital before MICU (OR:3.11) and length of stay in MICU (1.47). Mortality rate for VAP-patients was 80% and there was no statistically difference between the mortality rates of VAP and non-VAP patients.Median total cost of non-VAP patients in ICU was 2315 Euro, whereas it was 6308 Euro in VAP patients. Also, cost of ICU (253&908 Euro), antibiotics (230&810 Euro), laboratory (370&979 Euro) and clinical (371&878 Euro) were higher in VAP patients.

## Conclusion

The cost of VAP is approximately three-fold higher than non-VAP patients. Infection control standards should be assessed and rigorously reinforced in “limited-resources” countries.

## Disclosure of interest

None declared.

